# Supplementary material for: Lysyl-Oxidase Dependent Extracellular Matrix Stiffness in Hodgkin Lymphomas: Mechanical and Topographical Evidence
Source: Cancers (Basel). 2022 Jan 5;14(1):259. doi: 10.3390/cancers14010259 (PMC8750937; doi:10.3390/cancers14010259)
Supplement: Supplementary file 1 [file cancers-14-00259-s001.zip › cancers-1496078-supplementary.pdf]

cancers MDPI

Supplementary Materials

Lysyl-Oxidase Dependent Extracellular Matrix Stiffness in Hodgkin Lymphomas: Mechanical and Topographical Evidence

Massimo Alfano, Irene Locatelli, Cristina D'Arrigo, Marco Mora, Giovanni Vozzi, Aurora De Acutis, Roberta Pece, Sara Tavella, Delfina Costa, Alessandro Poggi and Maria Raffaella Zocchi

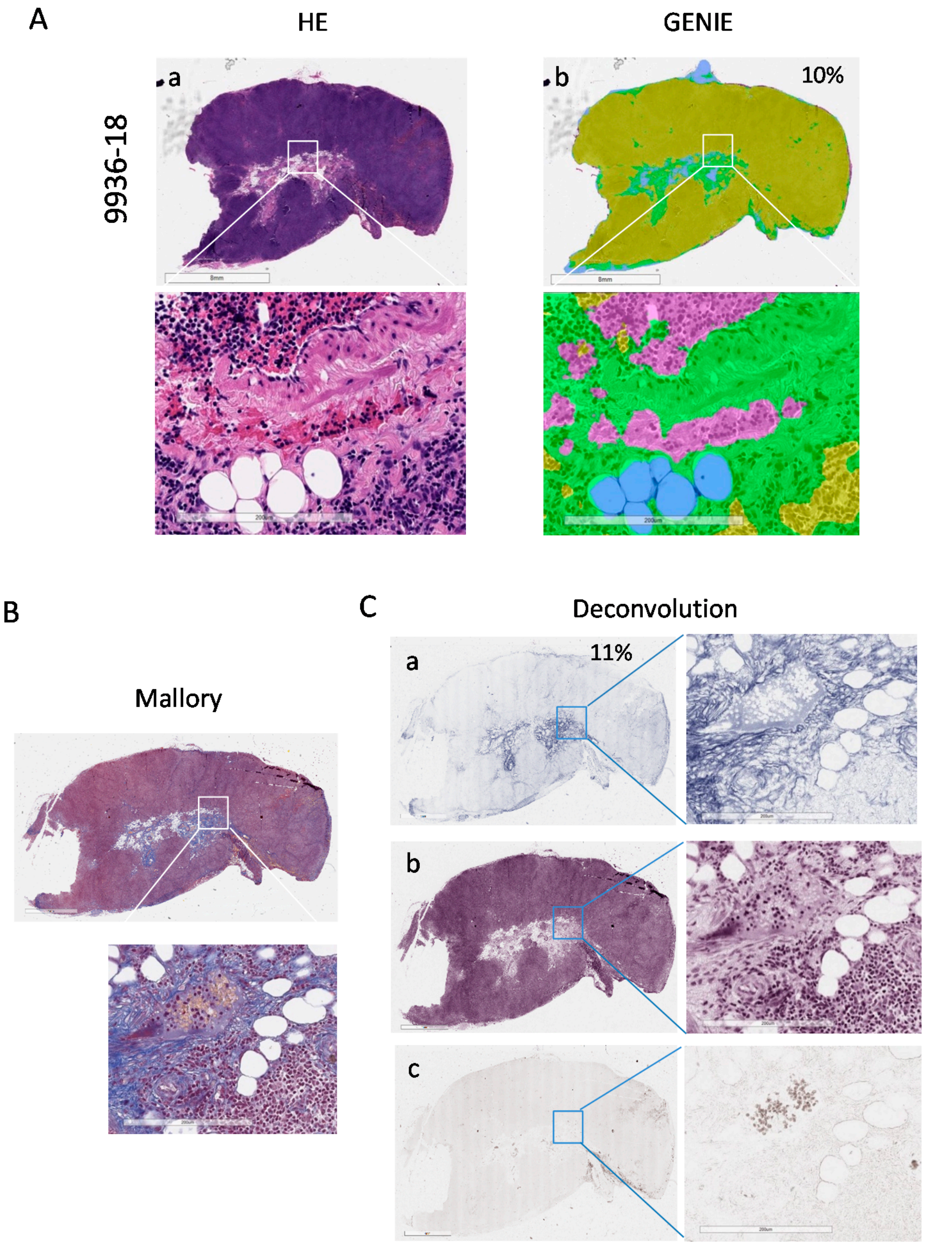

Figure S1. Digital pathology imaging of lymph node stroma. Digital images of lymph node (9936-18, NHL) sections were acquired using the Aperio ScanScope Slide program of the AperioAT2 Scanner (Leica Biosystem, Aperio Technologies) at 20× magnification. Panel (**A**). Subpanel a: one representative HE slide (square enlarged in the lower image); subpanel b: pseudocolor images analyzed by the Genie pattern recognition software tool (Leica Biosystems) used to determine the percentage

area of stromal compartment (square enlarged in the lower image) per slide. Green pseudocolor: stroma; pink and yellow pseudocolors: cellular compartments; light blue pseudocolor: fat. Stromal area percentage (number in the upper right picture) was adjusted to total tissue area (mm^2) in the image analyzed, ignoring the glass. Panel (**B**). Mallory trichrome images of the same lymph node as in A (9936-18) were captured with the AperioAT2 scanner at 20x magnification (square enlarged in the lower image) and analysed with the Aperio Image-Scope software. Blue: collagen fibers. Panel C. Layers of each virtual Mallory slide deconvoluted with the automated image analysis algorithm (Aperio version 9.1). Squares enlarged in the right images: blue color (a): collagen fibers. Dark violet (nuclei) and light violet (b): cellular compartment; light brown images (c): red blood cells. Stromal area percentage indicated in the upper left picture.

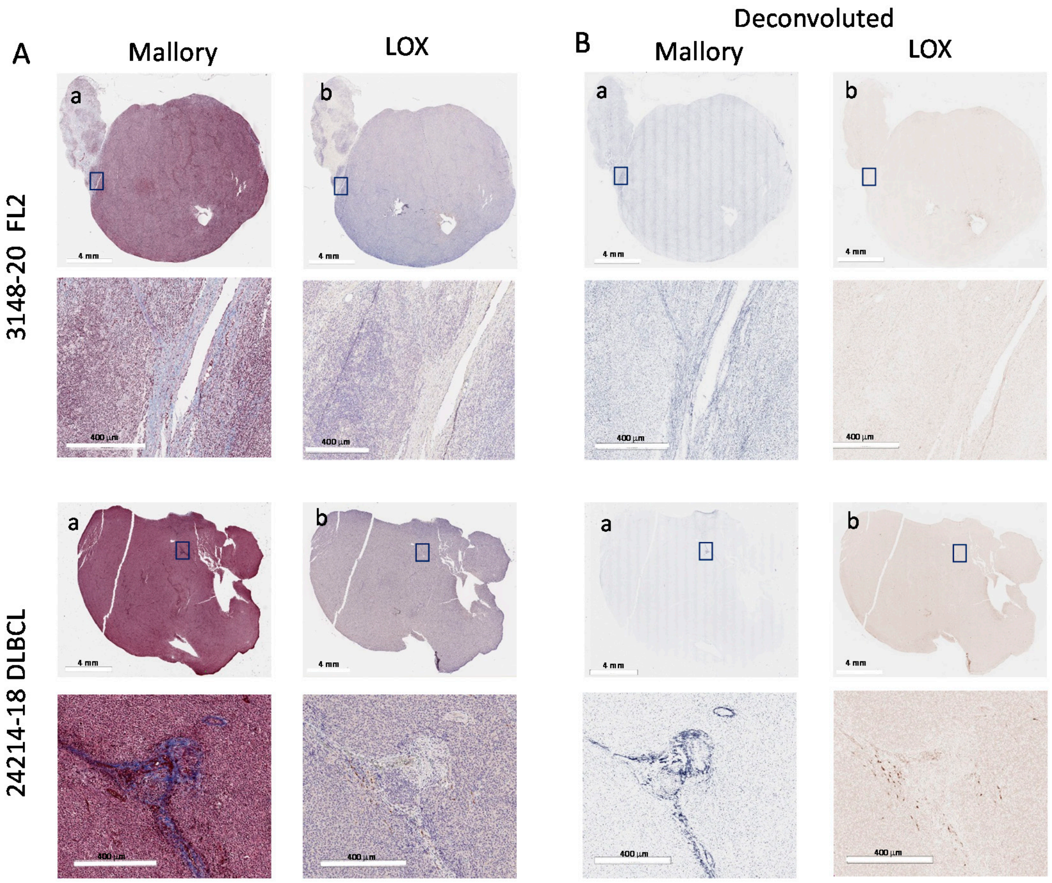

Figure S2. Digital pathology imaging of lymph node stroma in NHL FL2 and DLBCL. Digital images of lymph node sections (3148-20, FL2 and 24214-18, DLBCL) were acquired using the Aperio ScanScope Slide program of the AperioAT2 Scanner (Leica Biosystem, Aperio Technologies) at 20× magnification. Panel (**A**). Subpanels a: Mallory trichrome images. Subpanels b: IHC with the anti-LOX rabbit ab174316 mAb (Abcam). Panel (**B**). Layers of each virtual Mallory slides (a) or LOX IHC stained slides (b), deconvoluted with the automated image analysis algorithm (Aperio version 9.1). Enlargements of the squares depicted in each subpanel are also shown both in A and B.

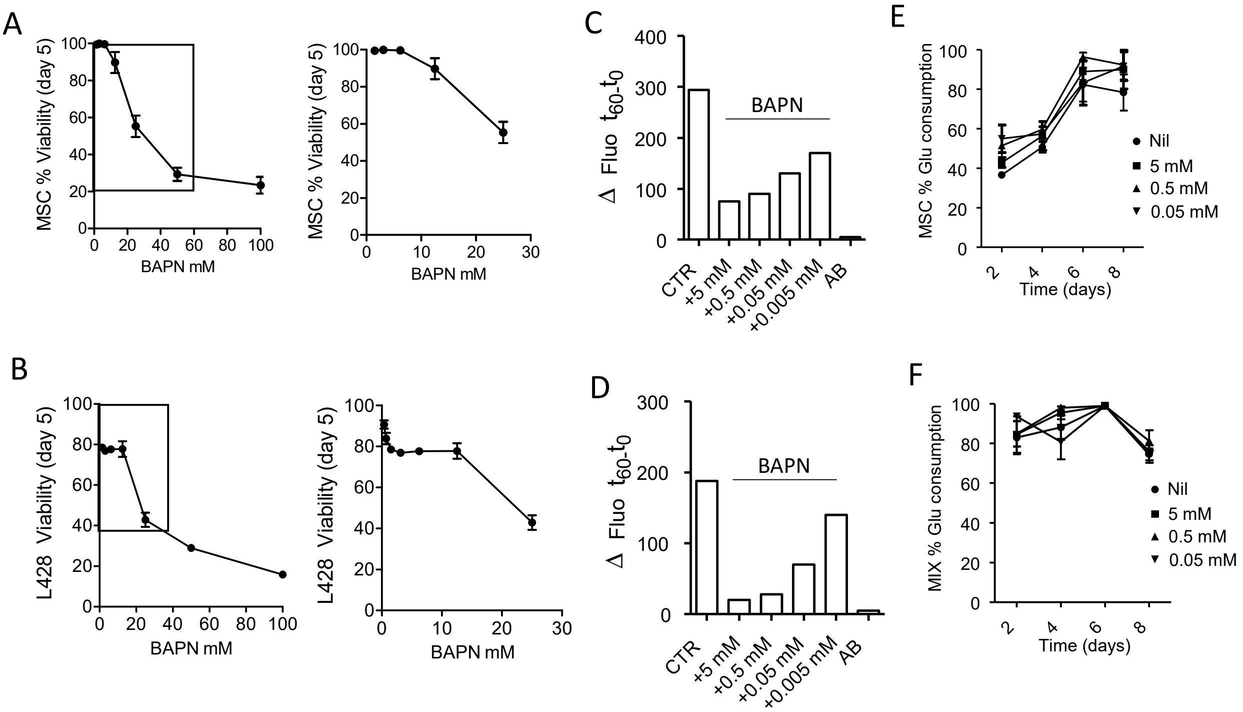

Figure S3. BAPN effects on LN-MSC and L428 HL cells. The LOX inhibitor BAPN was added at serial dilutions (100mmM to 0.05mM) on LN-MSC and L428 HL cells for 5 days. Panel (**A**): LN-MSC viability was determined with Crystal Violet Assay Kit (Biovision): the amount of crystal violet proportional to the amount of living cells was measured with the VICTORX5 multilabel plate reader at $O.D._{595nm}$ and data expressed as % viable cells. Panel (**B**): L428 cell viability was determined with the CellTiter-Glo® Luminescent Cell Viability Kit; luminescence was detected with the VICTORX5 reader and expressed as luminescence arbitrary units (a.u). Right graphs in A and B: enlargement of the rectangle in the left graphs. Panels (**C**) and (**D**): LOX activity evaluated by a fluorimetric assay in LN-MSC23274 (**C**) or L428 cells (**D**) in the presence of BAPN at the indicated concentrations. AB: buffer alone (background). Results are expressed as Δ mean fluorescence intensity (Δ Fluo arbitrary units, a.u.) measured at t60-t0. Panels (**E**) and (**F**): Glucose was measured in the SN recovered at the indicated time points from GelMA scaffolds repopulated with either LN-MSC23274 (E) or LN-MSC23274+L428 cells (MIX, F) co-cultures, untreated or treated with BAPN as above, with the D-glucose Assay Kit and referred to a standard curve. Data expressed as percentage glucose consumption vs glucose content in culture medium. A, B, D and F: Mean±SEM of 3 experiments performed in triplicate.
